# An Alternative Approach to Water Regulations for Public Health Protection at Bathing Beaches

**DOI:** 10.1155/2013/138521

**Published:** 2013-01-29

**Authors:** Amir M. Abdelzaher, Helena M. Solo-Gabriele, Matthew C. Phillips, Samir M. Elmir, Lora E. Fleming

**Affiliations:** ^1^NSF NIEHS Oceans and Human Health Center, University of Miami, Miami, FL 33149, USA; ^2^Department of Civil, Architectural, and Environmental Engineering, University of Miami, Coral Gables, FL 33146-0630, USA; ^3^Miami-Dade County Health Department, Miami, FL 33056, USA; ^4^Department of Epidemiology & Public Health and Marine Biology & Fisheries, University of Miami, Miami, FL 33136, USA; ^5^European Centre for Environment and Human Health, University of Exeter Medical School, Truro, Cornwall TR1 3HD, UK

## Abstract

New approaches should be considered as the US Environmental Protection Agency (EPA) moves rapidly to develop new beach monitoring guidelines by the end of 2012, as these guidelines serve as the basis by which states and territories with coasts along the oceans and Great Lakes can then develop and implement monitoring programs for recreational waters. We describe and illustrate one possible approach to beach regulation termed as the “Comprehensive Toolbox within an Approval Process (CTBAP).” The CTBAP consists of three components. The first is a “toolbox” consisting of an inventory of guidelines on monitoring targets, a series of measurement techniques, and guidance to improve water quality through source identification and prevention methods. The second two components are principles of implementation. These include first, “flexibility” to encourage and develop an individualized beach management plan tailored to local conditions and second, “consistency” of this management plan to ensure a consistent national level of public health protection. The results of this approach are illustrated through a case study at a well-studied South Florida recreational marine beach. This case study explores different monitoring targets based on two different health endpoints (skin versus gastrointestinal illness) and recommends a beach regulation program for the study beach that focuses predominately on source prevention.

## 1. Introduction

There is a growing health concern related to swimming in contaminated waters. Globally, each year, there are in excess of an estimated 120 million cases of gastrointestinal disease and in excess of an estimated 50 million cases of more severe respiratory diseases associated with swimming and bathing in wastewater-polluted coastal waters [1]. Since the 1950s, epidemiologic studies have been designed to evaluate the relationship between swimming in point source-impacted beaches and health risks (i.e., gastrointestinal disease, respiratory, eye, nose, and throat illnesses); they have concluded that symptoms for all these illnesses were increased in swimmers compared to nonswimmers [2, 3]. Outbreak reports from the CDC also confirm that illnesses in the USA are occurring from swimming in contaminated waters [4]. The excess illnesses associated with coastal water pollution can also result in substantial economic burdens. A study in Orange County, CA, estimated 3.3 million US dollars per year in excess illness costs for Newport and Huntington Beaches associated with bathing in marine waters [5].

Swimming-related illness is predominately associated with exposure to microbial pathogens, which may enter the water through point sources such as sewage outfalls. However, recent studies have demonstrated that swimming-related illnesses can occur at nonpoint source beaches [6, 7] and even possibly from beach sand contact [8–10]. These developments highlight the ongoing challenge to beach managers and public health professionals in monitoring the beach environment and protecting the health of its users [11, 12]. To address this challenge, we provide background information about existing regulations; describe a possible alternative approach given the existing regulations; and then apply this alternative approach to a well-studied South Florida recreational marine beach to illustrate one possible outcome of the alternate approach.

### 1.1. Current US Regulation

At the US federal level, jurisdiction over recreational waters is provided through the Clean Water Act (CWA). The CWA provides the US EPA with jurisdiction over two approaches for the control of water quality. First, it provides jurisdiction to regulate discharges of pollutants into the waters of the United States. Specifically, the CWA regulates discharges that represent point sources of pollution, that is, industrial, municipal, and other discharges via conveyances such as pipes or man-made ditches. Second, the CWA provides the authority to set point-of-use standards for surface waters. Waterbodies must not exceed contaminant levels beyond allowable values as determined by their “designated use” (swimming, bathing, surfing, water skiing, etc.). In most situations, the authority to issue discharge permits and to set designated use standards is delegated by the EPA to the states, thereby providing the states with the full authority to implement, that is, monitoring compliance, permitting, and enforcement.

**Figure 1:**
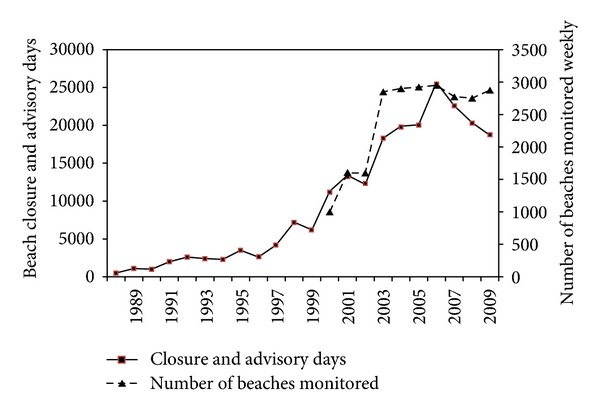
Number of beaches monitored and days of closures and advisories in the US [11, 14].

The Beaches Environmental Assessment and Coastal Health (BEACH) Act of 2000, an amendment to the CWA, sets forth beach water quality guidelines for 30 eligible states and territories with coasts along the oceans and Great Lakes. These guidelines include limits for *Enterococcus* in marine waters and for both *Enterococcus* and *E. coli* in freshwater. A single-maximum value, as well as a monthly geometric mean, is recommended by the Act [13]. While all 30 states affected by the BEACH Act follow these guidelines, many states employ unique additional sets of parameters to assess the safety of recreational waters (Table 1) [14]. In the U.S water monitoring includes measures at approximately 3,000 beaches for fecal indicator bacteria, resulting in 18,500 closures or advisory days in 2009 issued for recreational beaches [14] (Figure 1).

Seventeen of the 30 states under the BEACH Act preemptively close at least some of their beaches after significant rainfall. Twenty-eight states vary sampling based on season and sampling more often during the summer months. Twenty-two states vary the frequency of sampling based on usage and sample more often at beaches with higher human usage. Twenty-five states base the location of their sampling on usage or proximity to a possible source of contamination. Two states (Massachusetts and Hawaii) test waters for pharmaceutical chemicals that would be discharged with human sewage. Seven states have predictive models in effect for some of their beaches; many of which have proven to be more effective than the culture-based monitoring techniques [15]. Four states bordering the Gulf of Mexico close beaches after hurricanes. Texas monitors for *V. vulnificus* in addition to *Enterococcus* and Hawaii adds *C. perfringens* to their microbial testing. New York and Rhode Island utilize sanitary surveys in their monitoring efforts, taking into consideration environmental parameters (such as tidal stage, wild life, and seaweed). New Hampshire has an internet-based system in place where advisories are posted based on self-reported illnesses acquired from swimming at certain beaches [14]. Florida monitors its coastal beaches on a weekly basis year round using *Enterococcus* as the fecal microbe indicator. Florida applies the 2004 EPA's Recreational water Quality Criteria (RWQC) for a single sample and geometric mean to issue beach advisories [16].

### 1.2. International Regulations

International regulations include those of the World Health Organization (WHO) and the European Union (EU) [17, 18]. The WHO approach differs from the US traditional monitoring guidelines in that the susceptibility of the beach to fecal pollution (i.e., the likelihood of various fecal sources from reaching the water) is taken into account when determining the monitoring criteria [17]. Beaches without a known point source of human fecal pollution are allowed higher levels of fecal indicator microbes than ones which do.

The EU [18] regulates the sampling program (frequency of sampling, location of sampling, and time of sampling), including algae and/or their toxins as indicators of human health risk [19] and, most importantly, the requirement of “beach water profiles” for all beaches in the EU. The beach water profile is a critical addition to the regulation since it requires beaches to be assessed for physical, geographical, and hydrological conditions as well as potential pollution sources. These profiles are to be updated every 2 to 4 years depending on their history (i.e., whether determined to be excellent, good, sufficient, or poor) [18].

## 2. Methods, Alternative Approach to Current Beach Regulations

The proposed method is based upon an alternative approach which takes into consideration existing beach regulations. It is clear that the US regulations focus on contaminant discharges and monitoring for fecal microbe indicator levels, whereas international regulations rely more heavily on the allowable water quality levels, which for the WHO include an expectation of flexibility based upon susceptibility of the beach.

**Figure 2:**
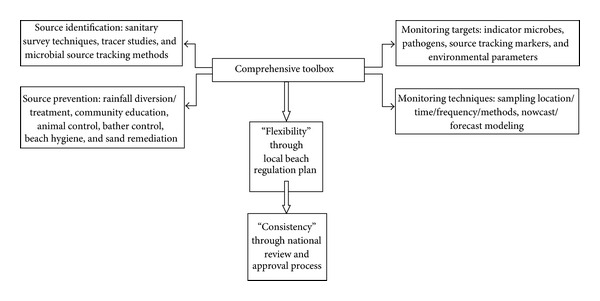
Comprehensive Toolbox within an Approval Process (CTBAP) Approach. This approach is based upon three steps which include: an inventory through the comprehensive toolbox (CTB) and two implementation principles to ensure “flexibility” and “consistency”. The inventory (a.k.a. comprehensive toolbox, CTB) consists of the methods and tools to address recreational water quality and include guidance for source identification and prevention along with guidance for monitoring targets and techniques. The principles of implementation are “flexibility” which is to be incorporated into the local beach regulation plan and “consistency” through a national review panel charged with assuring a consistent level of public health protection.”

Specifically, we propose the establishment of an effective criterion which we call the “Comprehensive Toolbox within an Approval Process, (CTBAP)” (Figure 2). The CTBAP is based upon three steps which include an inventory and two implementation principles. The inventory (a.k.a. comprehensive toolbox, CTB) consists of the methods and tools to address recreational water quality. These include guidance for source identification and prevention, along with guidance for monitoring targets and techniques. The two principles of implementation are “flexibility” which is to be incorporated into the local beach regulation plan and “consistency” to provide a consistent level of public health protection at the national level. A consistent national level of health protection implies that the monitoring targets provide the same level of risk, regardless of geographic location. So instead of setting the allowable indicator bacteria level at one consistent value, which is currently practiced through the US regulations, health risks would be set at a consistent level. The underlying theory behind this approach is to allow and expect for state, county, or even beach-specific flexibility, with a national approval mechanism for a nationally comparable level of human health protection. The methods and techniques in the alternative approaches would then be incorporated into the CTB.

This national CTB with initial organization at the federal level would create a resource which local teams (consisting of managers and scientists as well as other parties with expertise in the specific local conditions) may use to strategize the local beach management plan that would work best for them. A national CTB, in addition to providing options for monitoring targets and techniques, should also include guidance concerning sampling time, location, frequency, and method; predictive modeling (forecast and nowcast); information on how to communicate beach conditions to beach goers; sanitary survey methods; source tracking/tracer study methods; health risk study techniques to include quantitative microbial risk assessments, epidemiologic studies, self-reporting systems; pollution prevention methods via infrastructure, community education, animal control, and so forth. This CTB will need to be updated continuously by the national regulatory agency to keep up with the knowledge gained from new advances in the field of recreational water quality monitoring and pollution prevention.

Specifically to provide “flexibility” to state and local beach regulators, we recommend (1) an allowable range of targets based upon the susceptibility of the beach to fecal pollution as in the WHO approach and (2) an allowance for additional measures and controls as in the EU approach. We believe the concept of flexibility can also go further to include allowances for multiple lines of evidence to establish an overall safety level. For example, currently in recreational water quality monitoring, some states include multiple measures, but typically these measures are independent; if the threshold for either measure is exceeded, then an advisory is issued. Another component of the “flexibility” paradigm would be to allow for a safety assessment based upon integrated lines of evidence. An integrated line of evidence is consistent with the approach used for air quality standards. For example, for air quality standards, multiple constituents are measured, and collectively they are integrated to develop a “hazard” index, that is, Air Quality Index (AQI) [20]. A similar approach can be included as an option in the CTBAP process for developing standards for recreational bathing, where multiple lines of evidence can be used to collectively evaluate the probability of illness. These multiple lines of evidence can include measures of indicator microbes, results from modeling, weather conditions, measures of additional microbes (including possibly pathogens), and other factors.

The second principle, that of “consistency,” establishes a consistent health protection level across the country. One concern in providing too much flexibility to the states is that health protection will not be equal across the nation. The solution for this may be a national and diverse team of experts under a national agency (such as the US EPA) which could regularly review the proposed beach regulation plans of the individual states. Given the specificity of the beach, the current state of the science, and experiences as well as beach regulation plans of other beaches, this panel will either approve or recommend certain modifications to the beach regulation plan. This will allow for the development of a site-specific plan that is acceptable and comparable to other beach regulation plans. Therefore, the baseline used is not a single microbiological criterion, which may give a false sense of equal protection (since recommended indicators perform differently at different beaches and under different conditions), but a single team of expert reviewers who would assess health risk.

In order to implement such an approach, beach regulators should be given a reasonable amount of time to develop their beach regulation plans after the release of the CTB by the EPA. Until then, the traditional criteria may remain in effect. As the number of approved plans increases, a matrix may develop similar to that proposed by Boehm et al. 2009 [11]. The matrix as recommended by these authors would be based on certain factors (such as climate conditions and pollution sources) as well as any other factor that is deemed important by the state and/or local regulatory agency. Although this direction of beach regulation is recommended for the upcoming years and decades as science advances rapidly, such an approach will also be more difficult to apply than the traditional approach of beach regulation and requires initially a larger burden on both the local beach regulators and the EPA. Therefore, the actual implementation of such an approach will depend on when such an approach becomes feasible at the local and national levels.

## 3. Results, Outcomes from Applying the Recommended Approach

To illustrate some of the aspects of the recommended approach, a well-studied nonpoint source beach is used as a case study. Assuming the existence of a comprehensive toolbox as proposed above from which guidance may be derived, along with the local conditions known about this beach, a beach regulation plan may be developed. What follows is a discussion of the different aspects that may be included in such a beach regulation plan which would then be submitted to the national regulatory panel for review and approval. Given that the case study beach is well studied, the data from the actual beach may be used in order to guide the development of the proper monitoring techniques/targets and source identification/prevention approaches to be included in the beach regulation plan. Regulators for beaches not well studied may need to couple knowledge of their beach with any available studies from similar beaches (similar in terms of pollution sources, geographic region, beach use, resources, etc.) in order to develop their site-specific beach regulation plan.

### 3.1. Beach Water Profile

The case study beach is located on Virginia Key within Miami-Dade County, Florida, USA, geographically classified as “subtropical” with an average ambient temperature of 24.8°C. This beach is the only beach in the county that allows pets [21]. Given the fact that admission is free and that the beach is located in a central location accessible by many of the county's residents, the beach can become overcrowded especially on weekends and holidays, during hours when the beach is open (between dawn and dusk) [22].

Extensive evaluation of the vicinity of the study beach did not find contributions from point sources of pollution to this beach (such as sewage outfalls, failing lift stations, and cross connections of sewage with storm drains), or less obvious nonpoint sources (such as septic tanks) [23]. This beach is usually in compliance with regulatory monitoring criteria but periodically (i.e., 0.9 times per year averaged from 2002 to 2012) has been placed under a beach advisory due to microbial water quality violations (Florida Healthy Beaches Program Database).

At the study beach, indicator microbe levels vary significantly both spatially (location) and temporally (time) [24–26], as is frequently observed at other beaches [27–30]. Enterococci, the current microbial target recommended for marine waters and used by regulators at the study beach, varied based on the location of sampling, with higher levels observed near the shoreline and lower levels observed offshore. These spatial differences are hypothesized to be due to Enterococci release from the intertidal sand zone where microbes are believed to regrow and persist [31–33]. Temporal variability is driven at the site by a combination of tidal, rainfall, and solar radiation effects [31, 34, 35].

Given the extensive beach-specific data, it becomes clear that any monitoring target (i.e., such as Enterococci) should include a monitoring technique that considers both spatial and temporal variability for the study beach (Table 2). If Enterococci from the shoreline sand are considered to be related to human health, the monitoring technique should be designed so that this Enterococci signal would be the most obvious. This would mean sampling at locations and times where microbial concentrations are expected to be most elevated and during times that people may be exposed to those waters (i.e., during the morning, after peak hightide or rainfall events, and as close as possible to the shoreline). However, if a relationship between Enterococci and human health at this study beach is believed to be driven by offshore sources of Enterococci, such as inadvertent sewage spills, regulators may consider developing sampling strategies that target these sources. This would entail sampling at locations and times where microbial concentrations from sand sources are expected to be least (i.e., during noon and afternoon, after peak low tide, avoiding sampling after rain events, and sampling offshore possibly at waist deep waters) (Table 2). In this case, elevated Enterococci levels would indicate a new offshore and potentially hazardous pollution source. Currently samples are collected in waist deep water which is consistent with a focus on identifying offshore sewage impacts and avoiding the high background levels associated with the nonpoint sources that originate from the intertidal zone.

### 3.2. Monitoring Targets and Disease Endpoints

Monitoring targets can include microbial, chemical, hydrologic, or another indicator of a health risk, whereas disease endpoints can include gastrointestinal, skin, respiratory, eye, and ear illnesses. The most common disease endpoint evaluated traditionally for recreational swimming is gastrointestinal illness (GI). A common method to compare potential monitoring targets to disease end-points is through epidemiologic studies. This is accomplished through identifying the associations between human health outcomes and monitoring targets. In the vast majority of beaches in the USA, an epidemiologic study specifically for that beach is not available, and therefore epidemiologic studies at similar beaches or other methodologies such as quantitative microbial risk assessment (QMRA) [36] may be used to assist in identifying appropriate monitoring targets.

At the study beach, a randomized control epidemiology study with comprehensive water microbial and environmental monitoring was completed [6, 34, 37]. During the epidemiology study, an increased risk of self-reported skin, respiratory, and gastrointestinal illnesses were associated with recreational bathing [6]. Statistically significant associations were observed between (a) skin disease and Enterococci levels determined by membrane filtration, (b) skin disease and to 24-hour antecedent rainfall, and (c) respiratory illness and the inverse of water temperature [37]. Neither environmental conditions nor microbes evaluated in this study were found to statistically relate to self-reported gastrointestinal illness. However, the results of the study did indicate a possible pattern between gastrointestinal illness and both F^−^ coliphage and *Giardia *spp., both being detected on 4 of the 15 total study days (Table 3) [34], and both were detected on 3 of the 5 days characterized by the highest excess gastrointestinal illness (see the number represented by asterisk (*) in Table 3). Given the principle of “flexibility”, these targets, F^−^ coliphage and *Giardia *spp., should be thus considered as additions to the CTB that is used to assess water quality at this site.

With respect to disease end-points, we implement the concept of flexibility through the consideration of multiple health endpoints [34] (Table 2), which is based on measured health endpoints from more than one type of illness (e.g., gastrointestinal, skin, and respiratory). With this methodology, regulators are potentially guarding against a wider range of health impacts that may result from bathing, and hence from other pollution sources besides human sewage containing enteric pathogens. However, the multiple health endpoints, when applied to the data available for the study beach, were driven by the increased report of skin illnesses given their predominance relative to other illnesses. Therefore, for the case study beach, the multiple health endpoints approach may be considered where one microbial target would be used to assess risks from skin illnesses and another target would be used to assess gastrointestinal illness.

### 3.3. Source Identification and Prevention

Although monitoring is an important aspect of beach regulation, source identification followed by source prevention is the key to establishing the safety of the beach environment in a long-term sustainable manner. This focus is also consistent with the jurisdiction of the EPA through the CWA to minimize contaminant discharges. However, the beach profiled for the study site did not identify point sources of pollution. Instead, several nonpoint sources were identified including rainfall runoff [31], bather shedding [38, 39], dog feces [21], and sand diffusion [40, 41]. Such observations are not only observed at the study beach but also at many other beach sites throughout the USA and beyond [11, 15, 42]. All of these sources come in contact with the beach sand and/or are directly released from the beach sand. Furthermore, analysis of the beach sand at the site has identified potential pathogenic microbes including protozoa, helminthes, fungi, and the bacteria, *S. aureus* [43, 44].

The focus should be thus on remediation of sand sources through multiple means, such as controlling runoff [45] and solid waste control [46], limiting the number of pets, birds [47], and minimizing human shedding through the provision of showers [43].

To address the need to remediate nonpoint diffuse sources of contamination, various source identification techniques [48] need to be available to beach regulators in the CTB in order for them to include these techniques in their beach regulation plan. Sanitary survey approaches, tracer study techniques, and microbial source tracking methodologies need to be simplified and made more accessible to regulators through inclusion in the CTB. Once sources are identified, through the guidance of the CTB, beach regulators can conduct various source prevention techniques as part of their beach regulation plan.

## 4. Discussion and Conclusions

The complexity of the beach system, especially that of beaches not dominated by a point source of pollution [34], implies that more novel and comprehensive approaches will be needed in order to more effectively protect the health of bathers, while at the same time limiting overconservatism which may lead to unnecessary beach closures and economic loss [5]. The CTBAP proposes the development of a comprehensive toolbox which provides an inventory of potential monitoring targets, measurement techniques, and guidance for source identification and prevention. The implementation would be based upon principles of “flexibility” where states are encouraged to identify their own targets as they develop beach management plans and “consistency” which includes an assessment of the plans at the national level to ensure a consistent level of human health protection.

To that end, the specific recommendations for the study beach reviewed here focus on controlling discharges and providing more flexibility for the allowable levels at the point-of-use. The study beach's main pollution source as described above is the sand which serves as a reservoir for indicator and pathogenic microbes inoculated into the sand by anthropogenic- and animal-related sources. Therefore, the continued monitoring of the sand and remediation as needed are recommended to prevent the sand from becoming a source of microbes that constantly contaminates the water via environmental conditions such as tidal action and rainfall. This is in addition to the source prevention techniques mentioned earlier (i.e., runoff diversion and treatment and bather and dog source remediation and prevention) to preemptively limit sand and water pollution.

Within the confines of the current US regulations, it is understood that the EPA has limited authority, including limitations to regulating water only (as opposed to regulating beach sands). Within this limitation and given the need to control pollution sources, it is recommended that states, as part of their implementation plans, consider the inclusion of sand measures as part of their monitoring program. Ideally beach management plans should include a focus on maintaining sand quality as a means of improving water quality. Ideally the CTB, established at the federal level, would acknowledge sand as a potential source and ideally the CTB would also provide guidance for measuring, modeling, and remediating microbes from sand sources.

With respect to allowable levels at the point-of-use, no relationship was found at the study site between fecal indicator bacteria and gastrointestinal illness. Indicator levels at the site are strongly dictated by environmental conditions which may not be related to the pathogens of concern and hence the lack of health relation. However, given the potential association with skin illness, Enterococci should still remain in this beach's monitoring criterion. Therefore, the recommended criteria should include Enterococci, with the understanding that an elevation in values may indicate a potential skin illness risk as opposed to a gastrointestinal illness-related health risk. This should then be taken into account by regulators in determining whether or not to place beach advisories based on this indicator alone. If such advisories are placed, it is recommended that the risk communication strategy take into account the potential disease outcome (e.g., recommending freshwater showers after using the beach).

Enterococci would also be beneficial to evaluate potential sewage contamination from offshore sources; as such the sampling protocol should include sampling, as explained earlier, where levels would be expected to be low based on sampling time and location (i.e., sampling in waistdeep water during noon or afternoon). High levels in these situations should be taken seriously as they may indicate the influence of other offshore fecal sources of pollution (e.g., from moored boats) besides the expected dominant source of sand diffusion.

A study is also recommended for this beach to determine whether the addition of F^−^ coliphage and *Giardia *spp. may serve as indicators of gastrointestinal-related illness given the prior limited epidemiologic results, and hence their addition to the monitoring program. This may be accomplished through an approach other than traditional epidemiology studies such as quantitative microbial risk assessments. If further studies warrant their use, F^−^ coliphage may be added as a monitoring target along with Enterococci, while *Giardia *spp. may be used as a confirmatory target for the presence of gastrointestinal health risk. The proposed beach regulation plan for point-of-use monitoring would be similar to the existing sampling plan for this beach except with a different understanding of how to use Enterococci and interpret its levels, an emphasis on source prevention (e.g., minimize dog fecal contamination, runoff management, solid waste management, etc.), and potential supplementary monitoring (F^−^ coliphage and *Giardia *spp.) to assist in predicting gastrointestinal illness.

Implementation of the “Comprehensive toolbox within an Approval Process,” for this particular study site would greatly benefit from guidance with respect to minimizing impacts from sand sources, would benefit from guidance in assessing measures of F^−^ coliphage and *Giardia *spp., would benefit from guidelines for sample collection and processing, and would benefit from guidelines for assessing nontraditional disease end-points such as skin ailments. These components can be added to the CTB once approved through the federal level review board thereby ensuring a comparable national protection level through this panel.

The authors acknowledge that development of a CTBAP would require a considerable amount of additional resources. Evaluation of state proposals and incorporating the approved methods into a federally maintained CTB requires validation of alternative approaches on a continuous basis, along with consensus building and considerable engagement with the states and beach managers. Implementation of the CTBAP would thus require a considerable investment in time and resources by both federal and state governments, an investment that could payoff in the long-term through improvements in water quality and ultimately public health. As implied by Dwight et al., [5], excess illness costs associated with recreational swimming are on the order of many of millions of dollars per year for two California beaches alone. Considering all of the beaches nationally, the payoff could be substantial for investments aimed at improving methods for monitoring and improving water quality at recreational beach sites.

## Figures and Tables

**Table 1:** Number of states applying various beach monitoring techniques [14].

Regulatory practice	Number of states
Sampling location	
Ankle	2
Knee	15
Waist	13
Frequency of sampling based on	
Usage	22
Season	28
Location of sampling based on Usage or point source	25
States allowed to issue advisories	30
States allowed to issue closures	17
Presumptive rainfall standards	17
Predictive models used	5
Unique microbial indicators used	2
Chemical indicators used	2

**Table 2:** Possible sampling strategies for the study site. The choice of sampling time and place depends upon which source (shoreline or offshore) is considered to be more strongly associated with human health.

	Target background levels for study site	
	Highest background Enterococci levels	Lowest background Enterococci levels
Primary source of Enterococci that would be measured	Microbes from shoreline	Microbes from offshore sources not from shoreline sand
Sampling strategy	During morning, near peak high tide, or after rainfall and as close as possible to the shoreline.	During noon and afternoon, after peak low tide, avoiding rain events, and sampling offshore.

**Table 3:** Criteria of development based on GI illness health risk values based on microbial targets measured at study site during the beaches epidemiologic study [34].

	Sampling day	1	2	3	4	5	6	7	8	9	10	11	12	13	14	15
Microbe measure	Enterococci by MF (CFU/100 mL)	<2	15*	4*	15	13	99	109*	<2	3	29*	50	13	<2*	14	<2
	F^−^ coliphage (PFU/100 mL)	<0.3	1*	17*	<0.3	<0.3	<0.3	<0.3*	<0.3	<0.3	<0.3*	<0.3	**0.3**	0.3*	<0.3	<0.3
	Giardia (cysts/100 L)	**1.1**	<0.5*	<0.5*	<0.5	<0.5	<0.5	2.1*	<0.5	<0.5	2.3*	<0.5	<0.5	1.5*	<0.5	1

Excess illness (%)	GI	0.0	9.7*	4.8*	0.1	2.2	2.4	5.8*	0.2	0.5	4.2*	1.9	−0.1	4.1*	0.0	−4.9
	Skin	10.3	3.0*	4.8*	−2.3	4.2	8.6	8.3*	7.7	2.8	15.6*	11.5	5.1	5.9*	0.0	−1.1
	Respiratory	0.5	0.0*	5.1*	−4.7	0.0	0.0	5.9*	7.3	2.2	2.0*	2.0	0.0	−2.0*	0.0	0.0
	Cumulative	10.8	12.7*	14.6*	−6.8	6.3	10.9	20.0*	15.1	5.6	21.8*	15.4	5.1	8.0*	0.0	−6.0

*Corresponds to days with highest gastrointestinal illness.
